# Quantity discrimination by kittens of the domestic cat (*Felis silvestris catus*)

**DOI:** 10.1007/s10071-023-01784-z

**Published:** 2023-05-14

**Authors:** Péter Szenczi, Sandra Martínez-Byer, Jimena Chacha, Robyn Hudson, Oxána Bánszegi

**Affiliations:** 1 CONACYT—Instituto Nacional de Psiquiatría Ramón de la Fuente Muñiz, Unidad Psicopatología y Desarrollo, Calz. México-Xochimilco 101, 14370 Ciudad de México, Mexico; 2 Posgrado en Ciencias Biológicas, Unidad de Posgrado, Edificio A, 1er Piso, Circuito de Posgrados, Ciudad Universitaria, Coyoacán, 04510 Ciudad de México, Mexico; 3 Instituto de Investigaciones Biomédicas, Universidad Nacional Autónoma de México, AP 70228, 04510 Ciudad de México, Mexico

**Keywords:** Spontaneous quantity discrimination, Numerical abilities, Object file system, Analog magnitude system, Two-way food choice test

## Abstract

Quantitative abilities are well described in many species and in diverse life situations, including in the adult domestic cat. However, such abilities have been much less studied during ontogeny. In the present study we examined spontaneous quantity discrimination by pre-weaning age kittens in two-way food choice experiments. In Experiment 1, 26 kittens performed 12 trials with different ratios between the number of same-size food items. In Experiment 2, 24 other kittens performed eight trials with different ratios between the size of two food items. We found, in general, that the kittens discriminated between the different amounts of food and spontaneously chose the larger one, but that their choice was influenced by the ratio of difference. The kittens in Experiment 1 chose the larger number of same-size food items if the ratio was smaller than 0.4 and in Experiment 2 they chose the larger pieces of food if the ratio between the items was smaller than 0.5. Because the kittens’ choice was not influenced by the absolute number of food items or the numerical difference between them in Experiment 1, it suggests that their cognitive performance relied on an analog magnitude system rather than on an object file system during the quantity discrimination tasks. We discuss our results considering the ecological and social background of cats and compare it with the performance of previously studied species.

**Supplementary Information:**

The online version contains supplementary material available at 10.1007/s10071-023-01784-z.

## Introduction

It is well recognized that many animal species (Geary et al. 2015), including invertebrates (Bortot et al. 2021), possess the ability to discriminate quantities. Two different mechanisms, which are not necessarily mutually exclusive, have been proposed for quantity discrimination: the object file system (OFS) and the analog magnitude system (AMS) (Feigenson et al. 2004; Geary et al. 2015). The OFS relies on the animal’s working or short-term memory with which it tracks each individual object, and thus can deal with only small quantities and has an upper limit, most likely three or four objects. Alternatively, according to the AMS, choice is based on the ratio between stimuli rather than on exact quantities following Weber’s law which states that the accuracy of discrimination increases as the ratio between the quantities decreases. Thus, it can deal with large sets of objects, it has no upper limit and decision making can be fast (Feigenson et al. 2004; Gallistel and Gelman 1992).

Despite the large amount of research on quantity discrimination abilities in a number of species both in the field and laboratory only a few studies have been conducted during ontogeny, and most of what we know about the development of quantity discrimination comes from human research. In humans, accuracy of quantity discrimination improves from birth to adulthood: newborns are able to discriminate quantities only with a 1:3 ratio, whereas 6-, 10- and 72-month-old infants’ accuracy improves and they can distinguish numerosities with a 1:2, 2:3 and even a 5:6 ratio, respectively (reviews in Cantrell and Smith 2013; Halberda and Feigenson 2008). The precision of number assessment continues to improve across development, peaking at around 30 years of age in humans (Halberda et al. 2012). Additionally, some research suggests that children may use both systems; that they use the OFS when distinguishing between a small number of objects (Feigenson and Carey 2005; Feigenson et al. 2002), but can use the AMS too when discriminating between large quantities (Xu 2003; Xu and Spelke 2000; Xu et al. 2005).

The quantitative abilities of young individuals have only been studied in a few non-human species, but they seem to have a sense of number from birth and to increase in number acuity as they develop. Newborn guppies (*Poecilia reticulata*) can discriminate spontaneously between quantities but only if one of the stimuli is from the small quantity range (e.g. 2 vs. 5 or 3 vs. 8; Bisazza et al. 2010), although they can be trained to make large number discriminations (e.g. 7 vs. 14, Piffer et al. 2013). However, if the numerical information is excluded and the two stimuli differ only in their continuous property (cumulative area) guppies no longer discriminate between them (Miletto Petrazzini et al. 2014; Piffer et al. 2013). Findings of a recent study of young zebra fish (*Danio rerio*) are consistent with these results, at least in terms of number discrimination (Sheardown et al. 2022). In contrast with this, tadpoles of the two anuran species tested (*Bufotes balearicus* and *Pelophylax esculentus*) seem to have the capacity to discriminate between only small numbers (Balestrieri et al. 2019), suggesting that they rely more on the OFS, whereas fishes might operate on both.

Extensive studies in chicks of the domestic chicken (*Gallus gallus domesticus*), however, question the existence of the two systems, at least in this species. Since the chicks’ response time and accuracy is ratio dependent when they process both large (e.g. 10 vs. 20) and small numbers (e.g. 2 vs. 3) it suggests they rely only on the AMS (Rugani et al. 2013, 2014). To our knowledge the only mammalian study on the ontogeny of quantity discrimination, apart from children, was done in puppies of the domestic dog (*Canis lupus familiaris*), where puppies’ choices were found to be also ratio dependent, but where they discriminated ratios only below 0.25 (Miletto Petrazzini et al. 2020). In contrast, adult dogs spontaneously differentiate between a ratio of 0.6 (Miletto Petrazzini and Wynne 2016; Ward and Smuts 2007) and they can be trained to discriminate between objects with an area ratio of 0.8 (Rivas-Blanco et al. 2020).

Comparing cognitive abilities, in this case quantitative assessment, of closely related species of different ecological and social background during ontogeny might contribute to a better understanding of the possible evolutionary pressures operating on and the advantages of such abilities. In a previous study of the domestic cat (*Felis silvestris catus*) we found that adults spontaneously discriminate quantities when faced with different numbers or sizes of food items if the ratio is below 0.5 (Bánszegi et al. 2016), although they can be trained to distinguish between items even with a 0.67 ratio (Pisa and Agrillo 2009). In addition, adult cats differentiate between the number and size of live prey (Chacha et al. 2020). Two-month-old kittens spontaneously distinguished between food stimuli when they differed only in size but not in number (1 vs. 1, Bánszegi et al. 2021). However, they were tested with only two different ratios so far.

Thus, for comparative purposes we performed two experiments methodologically similar to those previously performed on adult cats (Bánszegi et al. 2016). In Experiment 1, our aim was to investigate whether, in a two-way spontaneous food-choice, kittens of domestic cats distinguish between different numbers of food pieces and whether they choose the larger number of items significantly more often than the array with the smaller number of items. In Experiment 2, using a similar method as in Experiment 1 and a different set of subjects, our aim was to test kittens’ spontaneous choice between two single food items of different size.

## General methods

### Subjects

We began by testing 62 mixed-breed kittens (26 male, 36 female) from 15 litters and 11 mothers (average litter size 4.13 ± 1.19 SD). However, 12 kittens (five male, seven female) were excluded from the analysis; eight exhibited a laterality bias, going to the same side in at least 11 of the 12 trials regardless of the stimuli presented, and four did not engage in the task (on three different occasions the kittens did not appear motivated to approach the food). The kittens were raised in different private homes in Mexico City and Cuernavaca, Mexico, nevertheless, the owners were asked to adhere to certain general housing conditions. Starting on the 4th postnatal week, kittens were provided with canned cat food twice daily and ad libitum dry cat food, as well as being nursed by their mothers. Water and sand boxes were always available. At the end of the study, kittens were given away as pets with the help of students and local veterinarians.

Testing began on postnatal week seven because by this age kittens’ sensory systems are well developed (Ehret and Romand 1981; Johns et al. 1979; Olmstead and Villablanca 1980; Olmstead et al. 1979; Rathjen et al. 2003), and they use all the gaits found in adult locomotion (Levine et al. 1980; Peters 1983), indicating sensory-motor maturation (Villablanca and Olmstead 1979). For three days before the start of testing, the kittens were habituated to the test equipment (see “Procedure” below) by putting the equipment in various positions in the kittens’ room for several hours each day and feeding them on the stimulus presentation sheet in the presence of their mother, littermates and experimenters.

### Food stimuli

Kittens can show marked individual differences in food preference (Becques et al. 2009; own observations; Bradshaw et al. 1996; Bradshaw et al. 2000; Wyrwicka and Long 1980). Thus, to ensure that subjects attended to the stimuli and were motivated to perform the tasks, prior to the start of testing the experimenter briefly offered each kitten an array of three types of food: their usual familiar canned food, a commonly used dry food, or canned tuna. The first food eaten by the kitten was then the stimulus used in all experiments with that individual (Bánszegi et al. 2021, 2016; Szenczi et al. 2018). Thirty, 19 and one kitten were tested with their familiar canned food, canned tuna and dry food, respectively. On all test days, prior to the first trial the experimenter gave the kitten a piece of the food used in the experiments to check that it was motivated to obtain it.

### Experimental setup

For comparative purposes we followed methods previously used to test adult cats (Bánszegi et al. 2016) and similar to methods originally used to test dogs and coyotes by Ward and Smuts (2007) and Baker et al. (2011), respectively, but adjusted slightly for kittens.

On each trial, the kittens were presented with two food arrays on two black square plastic sheets (12.5 cm × 12.5 cm, hereafter called ‘tray’). The two trays were presented 15 cm apart (distance between the inner edges of the sheets) on a larger matte gray plastic sheet (47 cm × 68 cm) placed on the floor to homogenize the background (Supplementary material 1b). We tested 12 quantity combinations in Experiment 1 (Supplementary material 1a) and nine in Experiment 2. Kittens were given a maximum of six trials per day. The sessions were on consecutive days. If some kittens were not motivated to participate during the sessions, we continued testing them on additional days until each had completed all the trials. All kittens finished the trials within four days. Each kitten was tested once with each combination.

### Procedure

The test procedure was similar to that reported in a previous study (Bánszegi et al. 2021). Kittens were tested in the morning around 8–9 am or in the afternoon around 5–6 pm and were deprived for four hours before testing (including the mother’s milk) in order for them to be sufficiently motivated to perform the tasks. While the kittens were removed from their room the experimenter arranged the test setup. She/he placed the trays, with the prearranged food portions, on the gray sheet and walked to the farthest point of the room, staying aligned with the middle of the gray sheet but facing away from it, and remained motionless. Then the second experimenter brought in one of the kittens and placed it in the starting position which was on a 25-cm high box to give the kittens a better, less-distorted view of the stimulus array. The box was aligned with the middle of, and 5 cm from the longer side of the plastic sheet. The second experimenter gently held the kitten from behind for three seconds before releasing it to make its choice. We defined “choice” as the kitten jumping down from the box, going to one of the trays and manipulating the food in any way (lick, eat, or touch with a paw). As soon as the kitten chose a food portion, the first experimenter removed the other tray and allowed the kitten to eat briefly from the chosen stimulus. To avoid inadvertent cueing, during the tests both experimenters looked straight ahead at the opposite wall or the ceiling until the kittens had made their choice. Additionally, the experimenter holding the kitten wore sunglasses and both experimenters wore masks covering their mouth and nose.

The order of presentation of each stimulus combination within each experiment and across subjects was randomly assigned using lottery cards. Each day the order of testing the kittens was also randomized. All trials were video recorded (Sony HDR-CX405) for later analysis.

### Data treatment and statistical analysis

Kittens’ overall (individual) performance was analysed using Sign tests. Binomial tests comparing the number of kittens choosing the larger or the smaller quantities were used to assess animals’ choices for each combination of stimuli. To test whether the subjects’ choice of the larger quantity varied with (1) the ratio between quantities, (2) the total number of food items in the test, (3) the numerical difference between quantities, or (4) the order in which the stimuli were presented we compared all possible general linear mixed models (binomial response variable, identity of kittens as random factor) with these factors using second order Akaike’s Information Criteria (AICC), and the model with the lowest AICC value was chosen (Anderson and Burnham 2004). We report AICC differences (ΔAIC_c_ = AICC_i_–AICC_best approximating_) to compare the different models for best approximating the data. We also calculated normalized Akaike weights (wi) for each model, which can also be interpreted as a measure of best approximation of the data set. Details of model selection can be found in Supplementary material 2. P values were extracted using Wald χ^2^ (type II) tests.

## Experiment 1: numerical quantity discrimination

In this experiment we investigated quantity discrimination by kittens in a spontaneous two-way food-choice task in which the paired stimulus arrays comprised different numbers of the same-size pieces of a preferred food. Consistent with the report on puppies and our previous findings in adult cats (see “Introduction”), we expected that kittens would choose the array with the larger number of items significantly more often than the array with the smaller number of items.

### Methods

We tested 26 kittens (14 male, 12 female) from eight litters and seven mothers. The food pieces were shaped using a cake decorating bag with a metal tip. The diameter of each piece was approximately 1 cm. Their position on the sheet was pre-determined—equidistant from the centre of the sheet and from each other—to ensure that the arrangement between each food item was consistent across trials and between kittens. We tested 12 quantity combinations. Eight of these were the same as those tested with adult cats (Bánszegi et al. 2016): 1 vs. 4 (ratio: 0.25), 1 vs. 3 (ratio: 0.33), 2 vs. 5 (ratio: 0.4), 1 vs. 2 (ratio: 0.5), 2 vs. 4 (ratio: 0.5), 3 vs. 5 (ratio: 0.6), 2 vs. 3 (ratio: 0.67), 3 vs. 4 (ratio: 0.75). Since a recent study on puppies reported the lack of differentiation between 1 vs. 4 pieces of food (Miletto Petrazzini et al. 2020), we added 1 vs. 6 (ratio: 0.17) and 1 vs. 9 (ratio: 0.11) to give the kittens a considerably easier tasks. We also added 2 vs. 6 (ratio: 0.33) and 4 vs. 6 (ratio: 0.67) comparisons to test whether the kittens would differentiate between larger numbers too. For the design see Supplementary material 1a.

### Results

Twenty-five of the 26 kittens (96.1%) chose the larger number more often than the smaller number across the twelve trials, while only one chose the larger and smaller numbers equally often. Performance of the kittens was significantly different from chance (one sample *t*-test *t*_25_ = 7.89, *P* < 0.001). We found no differences between the sexes (Wilcoxon signed-rank test *W* = 58, *n*_*female*_ = 12 *n*_*male*_ = 14, *P* = 0.172) on kittens’ overall performance.

**Fig. 1:**
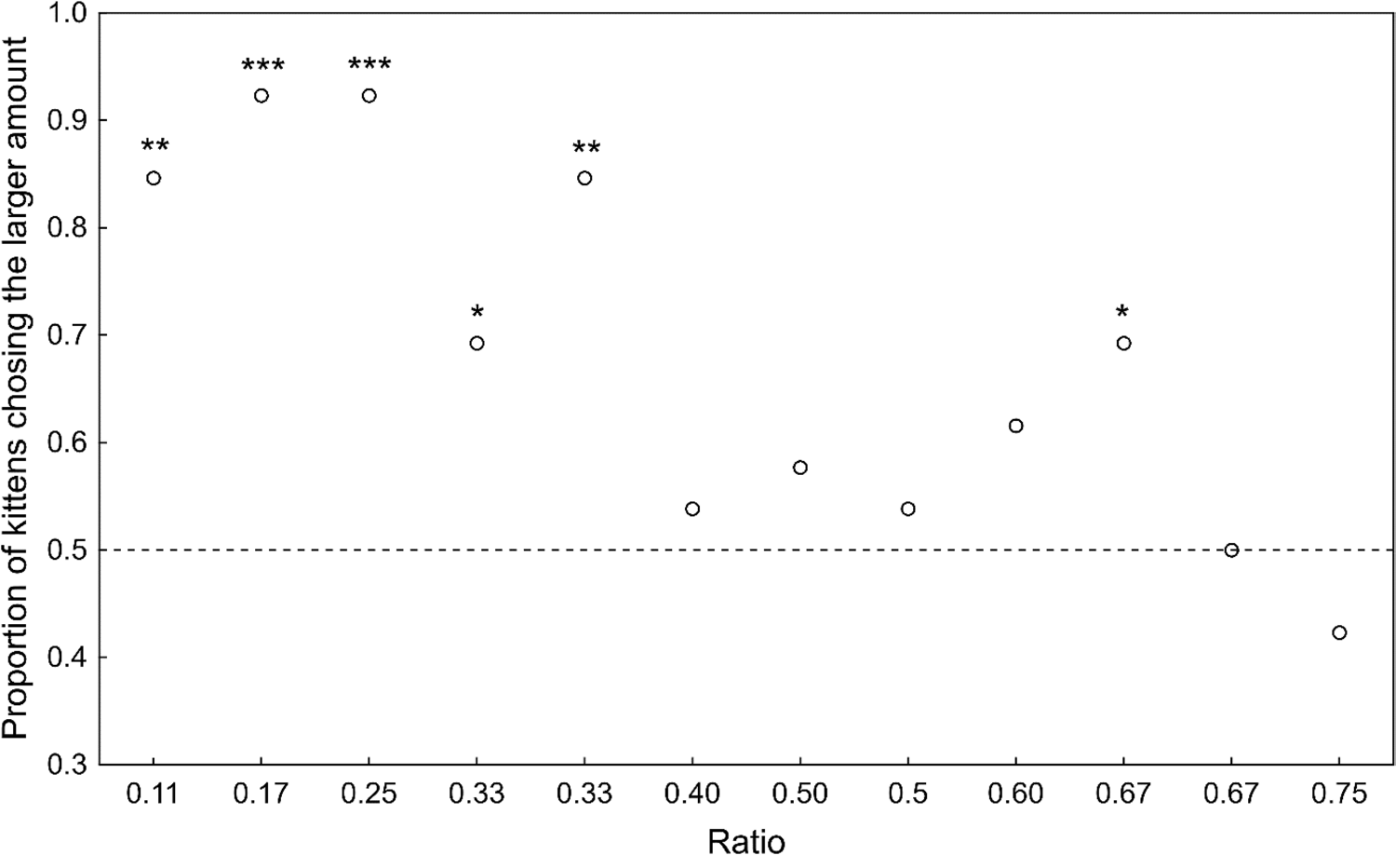
Proportion of kittens in Experiment 1 that chose the larger quantity of food items as a function of the ratio between the number of items in the stimulus array. *P < 0.05, **P < 0.01, ***P < 0.001. See text for details of statistical tests

Binomial tests of the kittens’ choice on each of the 12 combinations showed that only beneath a ratio of 0.4 did they reliably chose the larger number [binomial tests: *n* = 26, 1 vs. 9 (ratio: 0.11): 84.6%, *P* < 0.001; 1 vs. 6 (ratio: 0.17): 92.3%, *P* < 0.001; 1 vs. 4 (ratio: 0.25): 92.3%, *P* < 0.001; 1 vs. 3 (ratio: 0.33): 69.2%, *P* < 0.05; 2 vs. 6 (ratio: 0.33): 84.6%, *P* < 0.001; 2 vs. 5 (ratio: 0.4): 53.8%, *P* = 0.143; 1 vs. 2 (ratio: 0.5), 57.7%, *P* = 0.115; 2 vs. 4 (ratio: 0.5), 53.8%, *P* = 0.143; 3 vs. 5 (ratio: 0.6), 61.5%, *P* = 0.079; 2 vs. 3 (ratio: 0.67), 69.2%, *P* < 0.05; 4 vs. 6 (ratio: 0.67), 50.0%, *P* = 0.154; 3 vs. 4 (ratio: 0.75), 42.4%, *P* = 0.115; Fig. 1].

Comparing GLMMs using AICC with (a) ratio, (b) total number, (c) numerical difference between quantities and (d) order in which the stimuli were presented showed that the ratio between number of items had the strongest effect on the kittens’ choice; they were more likely to choose the larger number when the ratio between quantity combinations was small (GLMM ratio *β* = − 3.35, *χ*^*2*^ = 24.5, *P* < 0.001).

## Experiment 2: size discrimination

### Methods

As in Experiment 1, we followed the methods used in our previous study with adult cats (Bánszegi et al. 2016). In the present experiment we investigated quantity discrimination with 24 kittens (seven male, 17 female, from seven litters and six mothers) different from those in Experiment 1, in a spontaneous two-way food-choice task where the paired stimuli (1 vs. 1) comprised different sizes of two single pieces of food. Consistent with our previous finding (see “Introduction”), we expected that the kittens would choose the larger stimuli, at least until a certain ratio.

Food was shaped into a flat circle in the middle of the black tray using circular plastic moulds. We tested nine different size combinations where the difference was calculated based on the area of the food circles. Since from previous experiments we surmised that adult cats and kittens distinguish between different sizes of single food pieces below a 0.5 ratio (Bánszegi et al. 2021, 2016), this time we implemented a finer comparison and tested the following area combinations (1 = 3.14 cm^2^): 1 vs. 5 (ratio: 0.2), 1 vs. 4 (ratio: 0.25), 1 vs. 3 (ratio: 0.33), 2 vs. 5 (ratio: 0.4), 1 vs. 2 (ratio: 0.5), 3 vs. 5 (ratio: 0.6), 2 vs. 3 (ratio: 0.67), 3 vs. 4 (ratio: 0.75), 4 vs. 5 (ratio: 0.8).

### Results

Eighteen of the 24 kittens (75%) chose the larger quantity more often than the smaller across the nine trials, five (20.8%) of them chose the smaller quantity more often, while only one chose the larger and smaller quantity equally often. Performance of the kittens was significantly different from chance (one sample *t*-test *t*_23_ = 2.61, *P* < 0.05). We found no difference between the sexes (Wilcoxon signed-rank test W = 52.5, *n*_*female*_ = 12 *n*_*male*_ = 14, *P* = 0.95) on kittens’ overall performance.

**Fig. 2:**
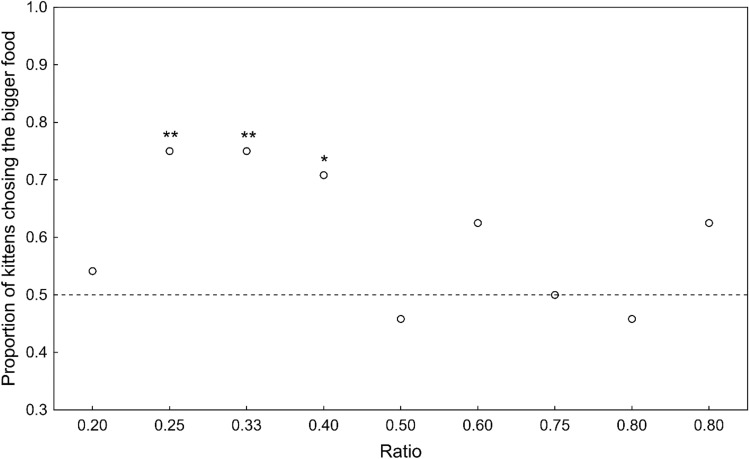
Proportion of kittens in Experiment 2 that chose the larger piece of food as a function of the ratio between stimulus sizes. *P < 0.05, **P < 0.01. See the text for details of statistical tests

Binomial tests of the kittens’ performance on each of the nine combinations showed that only beneath a ratio of 0.5 did they reliably chose the larger amount [binomial tests: *n* = 24, 1 vs. 5 (ratio: 0.2): 54.17%, *P* = 0.15; 1 vs. 4 (ratio: 0.25): 75.0%, *P* = 0.008; 1 vs. 3 (ratio: 0.33): 75.0%, *P* = 0.008; 2 vs. 5 (ratio: 0.4): 70.8%, *P* = 0.021; 1 vs. 2 (ratio: 0.5): 45.8%, *P* = 0.15; 3 vs. 5 (ratio: 0.60): 62.5%, *P* = 0.078; 2 vs. 3 (ratio: 0.67): 50.0%, *P* = 0.161; 3 vs. 4 (ratio: 0.75): 45.83%, *P* = 0.15; 4 vs. 5 (ratio: 0.80): 62.5%, *P* = 0.078; Fig. 2].

Kittens did not follow Weber’s law in this case, as the effect of ratio on performance was not significant. Although in general kittens’ responses showed a similar ratio-dependent pattern as in Experiment 1, the effect of ratio was not statistically significant (GLMM ratio *β* = − 0.96, *χ2* = 1.68, *P* = 0.19, order *β* = − 0.04, *χ2* = 0.50, *P* = 0.48).

## Discussion

Despite the fundamental role that quantitative abilities play in an animal’s life still little is known about the development of this. It has been studied only in a limited number of species and, to our knowledge, in only one non-human mammal, the domestic dog (Miletto Petrazzini et al. 2020). In the present study we investigated the spontaneous quantity discrimination of pre-weaning age kittens in two different experiments. We found, in general, that kittens discriminated spontaneously between quantities. In Experiment 1, we found that the kittens discriminated between different numbers of same-size food items if the ratio was smaller than 0.4, and in Experiment 2 we found similar results, that the kittens discriminated between two pieces of food of different size if the ratio between them was smaller than 0.5. These results are consistent with and similar to what we found previously in adult cats (Bánszegi et al. 2016) and kittens (Bánszegi et al. 2021) as well as with other carnivore species (review in Benson-Amram et al. 2017).

When we analyzed whether the total number, the numerical difference, the ratio between the quantities or the order of presentation of the stimuli influenced the kittens’ choice in Experiment 1, we found that only the ratio had a significant effect. The kittens were more likely to choose the larger quantity when the ratio between the number of stimuli was small. This suggest that kittens, similarly to adult cats, follow Weber’s law. Additionally, the lack of effect of the total number of presented food items or their numerical difference suggests that kittens use an AMS when they discriminate rather than an OFS, again similarly to adult cats (Bánszegi et al. 2016). When, in Experiment 2, the kittens had to choose between two pieces of food of different size, neither the order of presentation of the stimulus pair nor the ratio between them had a significant effect on their choice, although in Fig. 2 it can be seen that the kittens more reliably chose the larger portion of food when the ratio was smaller than 0.5 between the two stimuli except with the seemingly easiest task. The lack of effect of the order of presentation in both experiments suggests that there was no learning effect. The kittens’ percentage choice of the larger item dropped to chance level when they had to choose between the 1 vs. 5 times bigger food items. This preference for the smaller food portion or the avoidance of the larger one is very similar to what we found in adult cats (Bánszegi et al. 2016) and in our previous reports we have already discussed several, not necessarily mutually exclusive, possible explanations for this phenomenon (Bánszegi et al. 2016; Chacha et al. 2020).

Comparing the performance of the kittens in Experiment 1 to the performance of domestic dog puppies we can see that while puppies reportedly do not discriminate between 1 vs. 4 food items (Miletto Petrazzini et al. 2020), the kittens did so. Kittens possibly develop earlier a better or more precise system to differentiate between quantities. It is now generally accepted that the domestic cat evolved from its solitary-living ancestor (the wild cat, *Felis silvestris lybica*), and as an obligate predator, might have to develop this numerical ability sooner during ontogeny than social pack living dogs, whose ancestor is generally accepted to have been a social species (the gray wolf, *Canis lupus*). Although kittens might stay with their mothers after weaning for some extended period, probably they have to learn early how to assess the size of their potential prey to hunt safely and avoid possible injuries that could have serious or even fatal consequences (Chacha et al. 2020; cf. Panteleeva et al. 2013). Nevertheless, we should keep in mind that null results in the case of puppies do not necessarily mean the lack of ability to discriminate between stimuli. In spontaneous testing (as was the case for the puppies) motivation likely plays a key role and the difference between the 1 vs. 4 food items might not have been sufficiently motivating for the puppies, although motivating enough for the kittens to differentiate between the test stimuli (Miletto Petrazzini et al. 2020).

We can conclude that spontaneous quantity judgment and discrimination are part of the domestic cat’s natural behavioral repertoire from an early age. Kittens, like adult cats, will spontaneously choose larger quantities of food if they differ in number or size. However, if the size difference exceeds a certain ratio their differentiation drops to chance level. Reflecting on the present results in the context of current knowledge, we can conclude that the cognitive ability to discriminate between quantities probably emerges early during ontogeny in a variety of species which suggest that it may have been well conserved during evolution although also shaped by ecological and environmental factors.

## Supplementary Information

Below is the link to the electronic supplementary material.

Supplementary file1 (PNG 306 KB)

Supplementary file2 (DOCX 15 KB)

## Data Availability

The data that support the findings of this study are available from the corresponding authors upon reasonable request.
