# Supplementary figures and images for: Quantity discrimination by kittens of the domestic cat (Felis silvestris catus)
Source: Anim Cogn. 2023 May 14;26(4):1345–52. doi: 10.1007/s10071-023-01784-z (PMC10344966; doi:10.1007/s10071-023-01784-z)

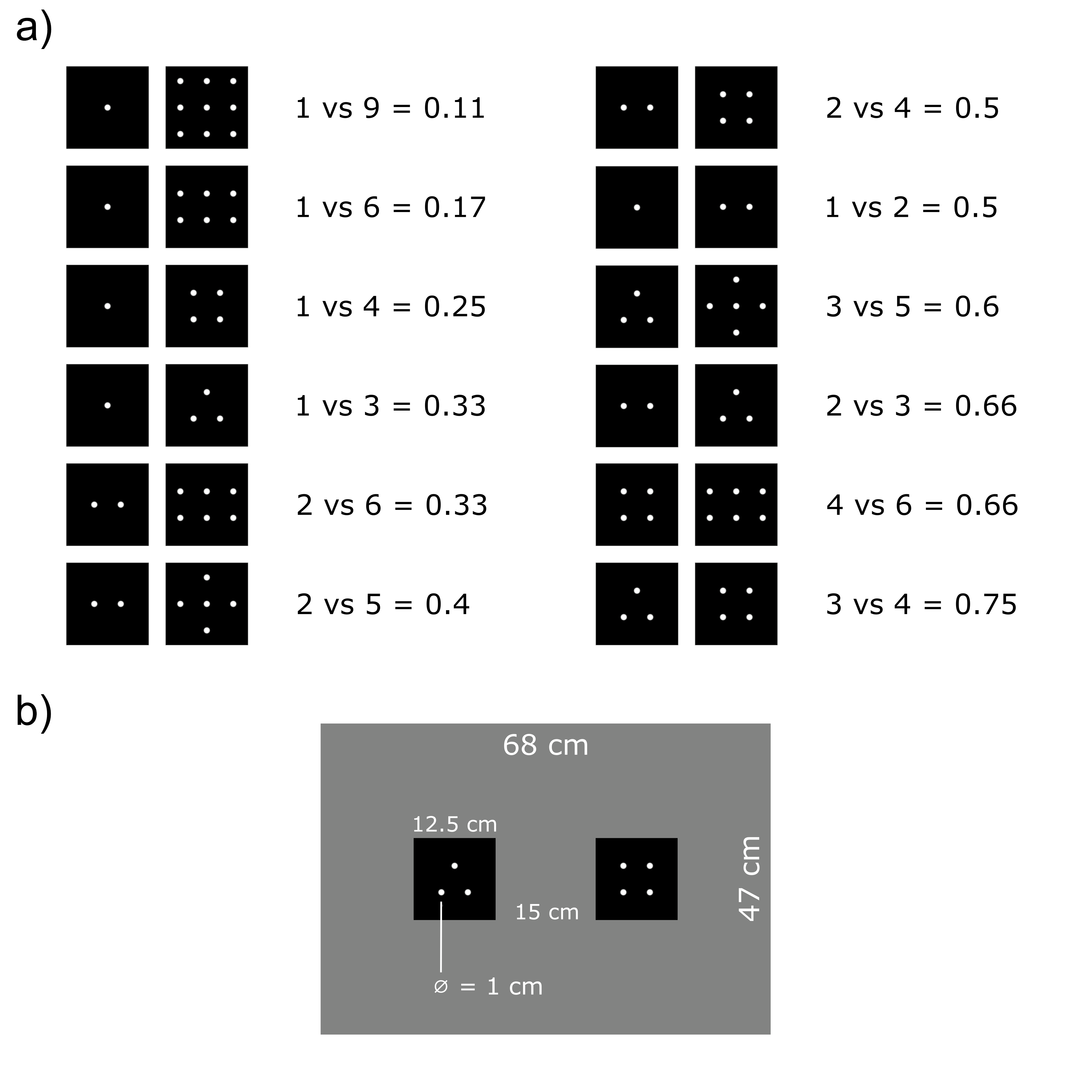

Supplement: Supplementary file 1 — Supplementary file1 (PNG 306 KB) [file 10071_2023_1784_MOESM1_ESM.png]
