# Supplementary material for: Quantity discrimination by kittens of the domestic cat (Felis silvestris catus)
Source: Anim Cogn. 2023 May 14;26(4):1345–52. doi: 10.1007/s10071-023-01784-z (PMC10344966; doi:10.1007/s10071-023-01784-z)
Supplement: Supplementary file 2 — Supplementary file2 (DOCX 15 KB) [file 10071_2023_1784_MOESM2_ESM.docx]

| Model terms | K | AICc | ΔAICc | w |
| --- | --- | --- | --- | --- |
| *Set of models for the quantity discrimination task* |  |  |  |  |
| **ratio** | **3** | **371.8311** | **0** | **0.3387** |
| ratio + order | 4 | 373.6824 | 1.8513 | 0.1342 |
| difference + ratio | 4 | 373.8098 | 1.9787 | 0.1259 |
| total number + ratio | 4 | 373.8799 | 2.0488 | 0.1216 |
| difference + total number | 4 | 374.8321 | 3.001 | 0.0755 |
| difference + ratio + order | 5 | 375.6959 | 3.8648 | 0.049 |
| difference + total number + ratio | 5 | 375.7243 | 3.8932 | 0.0484 |
| total number + ratio + order | 5 | 375.7482 | 3.9171 | 0.0478 |
| difference + total number + order | 5 | 376.6994 | 4.8683 | 0.0297 |
| difference + total number + ratio + order | 6 | 377.6002 | 5.7691 | 0.0189 |
| difference | 3 | 379.4514 | 7.6203 | 0.0075 |
| difference + ratio + order | 4 | 381.4939 | 9.6628 | 0.0027 |
| order | 3 | 398.9406 | 27.1095 | 0 |
| total number | 3 | 398.9675 | 27.1364 | 0 |
| total number + order | 4 | 400.9898 | 29.1587 | 0 |

The number of estimated parameters (*K*), AICc, ΔAICc (difference to the original model) and Akaike weights (*w*) are given. Selected model is in bold.
